# Festination Correlates with SNCA Polymorphism in Chinese Patients with Parkinson's Disease

**DOI:** 10.1155/2017/3176805

**Published:** 2017-03-05

**Authors:** Jinhua Zheng, Xinglong Yang, Quanzhen Zhao, Sijia Tian, Hongyan Huang, Yalan Chen, Yanming Xu

**Affiliations:** Department of Neurology, West China Hospital, Sichuan University, 37 Guo Xue Xiang, Chengdu, Sichuan Province 610041, China

## Abstract

The genetic basis of festination, a common motor symptom in Parkinson's disease (PD), remains unclear. Since polymorphism in the alpha-synuclein (SNCA) gene is associated with PD phenotype, we examined whether such polymorphism is also associated with festination. SNCA polymorphisms rs11931074 and rs894278 were genotyped in a consecutive series of 258 patients with PD, of whom 122 (47.3%) suffered festination. Univariate analysis revealed significant differences in genotype and minor allele frequencies at rs11931074 or rs894278 between patients with festination and those without it (all *p* < 0.05). Based on logistic regression, a GG or GT genotype at rs11931074 was associated with higher risk of festination among patients with PD (OR 2.077, 95% CI 1.111–3.883, *p* = 0.022), as was the TT genotype at rs894278 (OR 2.271, 95% CI 1.246–4.139, *p* = 0.007). Therefore, we conclude that festination is associated with polymorphism at rs11931074 or rs894278 among patients with PD.

## 1. Introduction

Parkinson's disease (PD) is the most common neurodegenerative movement disorder, affecting approximately 1% of people aged 65 or older worldwide [1]. Clinical manifestations of PD include motor deficits such as rigidity, slowness in movement (bradykinesia), postural instability, and a characteristic tremor at rest [1]. Motor symptoms of PD result from the selective loss of dopaminergic neurons in the pars compacta of the substantia nigra (SN) in the midbrain, as well as their axon terminals, which project to the dorsal striatum [2].

A neuropathological hallmark of PD is the presence of intraneuronal proteinaceous inclusions, termed Lewy bodies (LBs) or Lewy neurites. These structures are enriched in filamentous forms of the synaptic protein *α*-synuclein [3]. The gene that encodes the protein *α*-synuclein is alpha-synuclein (*SNCA*). Missense mutations within the* SNCA* gene or genetic duplication or triplication of the* SNCA* locus can lead to autosomal dominant forms of PD [4–6]. In addition to these rare* SNCA* gene mutations, some common genetic variations at the* SNCA* locus, including single-nucleotide polymorphisms (SNPs), are associated with higher risk of PD in the general population [7–9]. In Chinese populations,* SNCA* polymorphism has been associated with PD onset or progression. The T allele at rs11931074 and the G allele at rs894278 have been associated with significantly higher risk of PD [10], while the minor allele G at rs11931074 has been reported to reduce the risk of PD progression [10]. The TT genotype at rs11931074 may increase the risk of hyposmia [11].

Advanced PD commonly manifests with festination [12–14], which refers to the shortening of each step in a long gait sequence together with an increase in gait speed and involuntary forward-leaning of the trunk [15]. Festination in patients with PD may be associated with gait freezing and with PD duration or severity [13, 14, 16]. The genetic basis of festination is unclear, leading us in the present study to examine whether two loci linked to PD risk (rs11931074 and rs894278) are also linked to festination in Chinese patients with PD.

## 2. Methods

### 2.1. Study Population

The study protocol was approved by West China Hospital, Sichuan University. Written informed consent was obtained from all study participants. A consecutive sample of 258 Han Chinese patients was recruited from the PD clinic at our hospital between September 2009 and June 2014. All patients were diagnosed with sporadic PD based on Queen Square Brain Bank Criteria [17]. Patients were excluded from the study if they had other neurodegenerative disorders, severe medical illness, a history of schizophrenia, or bipolar disorder. Patients were also excluded if they had at least one relative with PD.

Patients were divided into those with festination (defined as described [15]) and those without it. Patients were classified as showing festination if it was observed by experienced neurologists during a visit to our PD clinic or if the patient, family member, or caregiver reported that it had occurred more than once during the previous week anywhere outside the hospital.

### 2.2. Clinical Assessment

We collected patient data on age, sex, and symptoms at PD onset, PD duration, and Hoehn and Yahr stage of PD severity [18]. Primary onset symptoms were classified as tremor or as akinetic-rigid (bradykinesia or rigidity), based on patient self-report. If the patient experienced tremor as well as akinetic-rigid symptoms at PD onset, he or she was asked to indicate which predominated.

### 2.3. SNCA Genotyping

Genomic DNA was obtained from peripheral leukocytes using classical phenol-chloroform extraction. The single-nucleotide polymorphisms (SNPs) rs11931074 and rs894278 in the* SNCA* gene were genotyped by the Shanghai Biowing Applied Biotechnology Company using the ligase detection reaction [19]. To ensure genotyping accuracy and reliability, genotyping technicians were blinded to sample identity, and 20% of samples were regenotyped by a different technician selected at random. The replication rate was 100%.

### 2.4. Statistical Analysis

Statistical analyses were performed using SPSS 16.0 (IBM, Chicago, IL, USA). Intergroup differences were assessed for significance using Student's *t*-test in the case of continuous variables or the chi-squared or Fisher's exact tests in the case of categorical variables. Logistic regression was used to test the association between SNPs and festination in PD. Results of multivariate analyses were presented as odds ratios (ORs) with 95% confidence intervals (CIs) and *p* values. All tests were 2-sided, and the threshold of significance was *p* < 0.05.

## 3. Results

The study involved 258 patients with PD, of whom 122 (47.3%) suffered festination, which was reported by the patient, family member, or caregiver in 113 cases (93%) or observed directly by neurologists in the remaining 9 cases (7%). Genotype frequencies at rs11931074 and rs894278 were consistent with Hardy-Weinberg equilibrium across all patients. Patients with festination were significantly older, they had suffered PD longer, and their PD was at a more advanced Hoehn and Yahr stage (Table 1). Univariate analysis suggested that polymorphism at rs11931074 affected risk of festination according to a dominant genetic model, while polymorphism at rs894278 affected risk according to a recessive model.

Next we performed logistic regression with the following covariates: sex, age, age and symptoms at PD onset, PD duration, Hoehn and Yahr stage (≤2 versus >2), genotype at rs11931074 (GG+GT versus TT), and genotype at rs894278 (TT versus GG+GT). Variables were selected using a forward logistic regression procedure. The analysis identified the following associated factors (Table 2): PD duration, onset symptom, Hoehn and Yahr stage, GG/GT genotype at rs11931074, and TT genotype at rs894278.

## 4. Discussion

This study provides the first evidence that, at least among Han Chinese patients with PD,* SCNA* polymorphism significantly increases risk of festination, specifically the GG/GT genotype at rs11931074 and TT genotype at rs894278. Our data also indicate that occurrence of festination is related to PD duration, Hoehn and Yahr stage, and onset symptoms. Further study is needed to clarify the molecular mechanisms behind these apparent associations.

Our data associate the T allele at rs11931074 and the G allele at rs894278 with lower risk of festination, while the same alleles have previously been linked to greater risk of PD in Han Chinese [10]. These results are not contradictory, since one study examined risk of PD and the other examined risk of festination in patients with the disease.

Our finding that longer disease duration is associated with higher risk of festination is consistent with results of studies in Israeli patients [14] and Han Chinese patients [13]. Our finding that more severe PD is associated with higher risk of festination is also consistent with both of those studies [13, 14]. A greater proportion of patients in our study had festination than in those two previous studies, perhaps reflecting differences in how patients were classified as experiencing festination or not.

Our data suggest that patients who experience bradykinesia or rigidity as the primary symptoms at PD onset are more likely to experience festination than patients with other onset symptoms. This is, to our knowledge, the first report of such an association, so this result should be verified in studies with larger samples and other ethnic groups.

Since nearly all cases of festination in our study were based on patient or caregiver report, our study may be affected by recall bias given the infrequent, episodic nature of festination. In addition, we did not systematically analyze possible nongenetic confounders, such as medication history or gait freezing. Nevertheless, we were able to show that the association between festination and* SNCA* polymorphism remained after adjusting for possible confounders of sex, age, and onset age. While we were able to observe an association between festination and disease duration, consistent with previous studies, we did not collect data allowing us to explore whether festination correlated with disease duration prior to onset of festination, which may help determine whether festination is linked to genetic variation per se or simply to longer disease progression and development of motor symptoms. Future studies should examine this question. Lastly, our patients came from a single medical center, albeit the largest one in western China that draws patients from Sichuan and neighboring provinces.

## 5. Conclusions

Despite these limitations, our study is the first to provide evidence that festination is associated with* SNCA* polymorphism at rs11931074 or rs894278. Our results should be verified and extended in larger studies, preferably involving multiple sites and ethnic groups.

## Figures and Tables

**Table 1:** Characteristics of Han Chinese patients with Parkinson's disease in the presence or absence of festination.

Variable	Festination (*n* = 122)	No festination (*n* = 136)	*p*
Males	78 (63.9)	75 (55.1)	0.151
Mean age, yr	64.8 ± 9.2	59.3 ± 11.9	0.000
<60	36 (29.5)	59 (43.4)	0.021
≥60	86 (7.05)	77 (56.6)	
Mean age at onset, yr	58.9 ± 10.2	55.9 ± 11.9	0.033
<55	44 (36.1)	54 (39.7)	0.548
≥55	78 (63.9)	82 (60.3)	
Disease duration ≥ 5 yr	63 (51.6)	28 (20.6)	0.000
Onset symptoms			0.129
Tremor	63 (51.6)	83 (61.0)	
Akinetic-rigid	59 (48.4)	53 (39.0)	
Hoehn and Yahr stage ≤ 2	38 (31.1)	82 (60.3)	0.000
rs11931074			
Genotype frequencies			0.015
GG	24 (19.7)	16 (11.8)	
GT	65 (53.3)	61 (44.9)	
TT	33 (27.0)	59 (43.4)	
MAF (G)	113 (46.3)	93 (34.2)	0.005
Dominant model (GG + GT)	89 (73.0)	77 (56.6)	0.006
Recessive model (GG)	24 (19.7)	16 (11.8)	0.080
rs894278			
Genotype frequencies			0.003
TT	61 (50.0)	40 (29.4)	
GT	51 (41.8)	78 (57.4)	
GG	10 (8.2)	18 (13.2)	
MAF (G)	71 (29.1)	114 (41.9)	0.002
Dominant model (TT+GT)	112 (91.8)	118 (86.8)	0.194
Recessive model (TT)	61 (50.0)	40 (29.4)	0.001

Results are shown as mean ± SD or *n* (%).

MAF: minor allele frequency.

**Table 2:** Logistic regression to identify factors associated with risk of festination in Han Chinese patients with PD.

Factor	*p*	OR	95% CI
Disease duration < 5 yr	0.000	0.224	0.116–0.435
Tremor as onset symptom	0.019	0.505	0.285–0.895
Hoehn and Yahr stage ≤ 2	0.007	0.439	0.242–0.798
rs11931074 (GG/GT)	0.022	2.077	1.111–3.883
rs894278 (TT)	0.007	2.271	1.246–4.139
